# Bacteriophages Contribute to the Spread of Antibiotic Resistance Genes among Foodborne Pathogens of the *Enterobacteriaceae* Family – A Review

**DOI:** 10.3389/fmicb.2017.01108

**Published:** 2017-06-20

**Authors:** Anna Colavecchio, Brigitte Cadieux, Amanda Lo, Lawrence D. Goodridge

**Affiliations:** Food Safety and Quality Program, Department of Food Science and Agricultural Chemistry, McGill University, Ste-Anne-de-BellevueQC, Canada

**Keywords:** bacteriophage, transduction, antibiotic resistance, foodborne pathogens, horizontal gene transfer, *Escherichia coli* O157:H7, non-typhoidal *Salmonella*, environment

## Abstract

Foodborne illnesses continue to have an economic impact on global health care systems. There is a growing concern regarding the increasing frequency of antibiotic resistance in foodborne bacterial pathogens and how such resistance may affect treatment outcomes. In an effort to better understand how to reduce the spread of resistance, many research studies have been conducted regarding the methods by which antibiotic resistance genes are mobilized and spread between bacteria. Transduction by bacteriophages (phages) is one of many horizontal gene transfer mechanisms, and recent findings have shown phage-mediated transduction to be a significant contributor to dissemination of antibiotic resistance genes. Here, we review the viability of transduction as a contributing factor to the dissemination of antibiotic resistance genes in foodborne pathogens of the *Enterobacteriaceae* family, including non-typhoidal *Salmonella* and Shiga toxin-producing *Escherichia coli*, as well as environmental factors that increase transduction of antibiotic resistance genes.

## Introduction

The World Health Organization (WHO) estimates that in 2010, foodborne illnesses affected 600 million people and caused 420,000 deaths globally (World Health Organization [WHO], 2015). In Canada, 4 million cases of foodborne illnesses, 11,600 hospitalizations and 238 deaths are estimated to occur annually (Thomas et al., 2015). In the United States, 47.8 million cases of foodborne illness are estimated to occur annually with 127,839 hospitalizations and 3,037 deaths (Scallan et al., 2011). The economic impact of foodborne illness in the USA is estimated at approximately $10–83 billion (USD) annually (Mclinden et al., 2014). A major contributor to the costs associated with foodborne disease is healthcare costs. The continued emergence of antibiotic resistance, and especially multidrug resistance, among foodborne pathogens may contribute to unsuccessful treatment outcomes, thereby increasing costs associated with foodborne disease. For example, in the United States, antibiotic resistant foodborne infections cause 430,000 illnesses annually (Centers for Disease Control and Prevention [CDC], 2015a). Therefore, there is a growing need to better understand the mechanisms, frequency, reservoirs, and vectors governing the transfer of antibiotic resistance determinants in major foodborne pathogens in hopes of controlling the dissemination of antibiotic resistance.

Non-typhoidal *Salmonella* is the most prevalent bacterial foodborne pathogen resulting in hospitalization in the western world (Thomas et al., 2015). Shiga toxin-producing *Escherichia coli* (STEC) are also important causes of foodborne illness that frequently result in hospitalization (Majowicz et al., 2014). Outbreaks linked to these pathogens have been associated with various foods. *Salmonella* outbreaks have been linked to contaminated poultry, eggs, cheese, ice cream, fresh produce, and chocolate, while STEC outbreaks have been primarily linked with fresh produce and ground beef (Kapperud et al., 1990; Voetsch et al., 2004; Werber et al., 2005; Centers for Disease Control and Prevention [CDC], 2015b; Herman et al., 2015).

In 5% of gastroenteritis cases caused by NTS, the patient will develop bacteremia or other extraintestinal complications such as urinary tract infections, pneumonia, endocarditis, meningitis, and cellulitis (Eng et al., 2015). In the 1980s, NTS was treated with ampicillin, chloramphenicol and TMP-SMZ but by the 1990s widespread resistance emerged (Acheson and Hohmann, 2001). Currently, fluoroquinolones and third-generation cephalosporins are mainly administered but resistance to these antibiotics is also emerging (Eng et al., 2015). Even more concern materialized when multidrug resistant strains of *Salmonella* Typhimurium phage type DT104 emerged. This strain has been associated with poultry, cattle, and swine, and is defined by ACSSuT (Threlfall, 2000). Data from the National Antimicrobial Resistance Monitoring System (NARMS) indicated that 8.5% of NTS isolated from humans between 1999 and 2004, and 3.8% of retail meat isolates from 2002 to 2004 had the ACSSuT phenotype and *Salmonella* Typhimurium and *Salmonella* Newport were the most common serovar with the penta-resistance pattern (Whichard et al., 2010). In fact, *Salmonella* was the most prevalent foodborne pathogen involved in antibiotic resistant outbreaks between 1973 and 2011, and *S.* Typhimurium was the most frequent serovar (Dewaal and Grooters, 2013).

In contrast, antibiotic treatment for STEC is controversial since some studies have observed certain classes of antibiotics to be effective while others increase the production of Stx due to the induction of Stx carrying prophages, which could trigger complications such as hemolytic uremic syndrome (HUS) (Kurioka et al., 1999; Bielaszewska et al., 2012). Still, antibiotic resistance among STEC strains has significantly increased since the first identification of STEC as a foodborne pathogen in 1982 (Kim et al., 1994; Meng et al., 1998; Galland et al., 2001; Schroeder et al., 2002; Vidovic and Korber, 2006), likely due to overuse of antibiotics in food producing animals such as ruminants, which are the animal reservoir for STEC (Hunt, 2010). For example, in 1988, one study indicated that 2.9% of *E. coli* O157:H7 isolates (the predominant STEC serotype, accounting for the majority of STEC cases in North America) from human cases were resistant to antibiotics (Ratnam et al., 1988). As of 2007, 79.8% of *E. coli* O157:H7 isolates from bovine and human feces, bovine milk products, ground beef, and cider carried one or more antibiotic resistance genes (Srinivasan et al., 2007). Although high percentages of antibiotic resistance have not been historically associated with STEC, additional studies support the increasing presence of antibiotic resistance in *E. coli* O157:H7 isolates. For example, Ferreira et al. (2015) isolated 90 *E. coli* O157 isolates from health sheep, and observed that 75 of the isolates (83.3%) were resistant to at least one antibiotic. In another study, antibiotic profiles of 95 fecal isolates of *E. coli* O157:H7 collected from two commercial dairy farms in South Africa demonstrated that *bla*_ampC_ (90%), *str*A (80%), *tet*A (70%), *bla*_CMY_ (70%), and *bla*_CTX-M_ (65%) were predominant (Iweriebor et al., 2015). El-Shatoury et al. (2015) also demonstrated that of 44 *E. coli* O157:H7 isolates, 100% were resistant to amoxicillin and 77% were resistant to clarithromycin.

Horizontal gene transfer mechanisms responsible for the increased spread of antibiotic resistance to foodborne bacterial pathogens have been well studied. Conjugation, transformation, and transduction are the primary mechanisms by which dissemination of antibiotic resistance genes occurs (von Wintersdorff et al., 2016). The notion that phage mediated transduction is a major driver of horizontal transfer of antibiotic resistance genes between foodborne pathogens, as well as from the environment to animals and humans, is increasingly becoming recognized. Phages are the most abundant organism in the biosphere, and are found in diverse environments including oceans, lakes, soil, urban sewage, potable and well water and plant microbial communities (Clokie et al., 2011). Antibiotic resistance genes are often found on various MGEs, such as plasmids, genomic islands and transposons, and, as such, can be horizontally transferred by phage transduction. This review summarizes the scientific literature in which horizontal transfer of antibiotic resistance genes by phage-mediated transduction has been described, with a specific focus on the role that phages play in horizontal transfer of antibiotic resistance genes among foodborne pathogens, including STEC O157:H7 and *Salmonella enterica*, as well as between environmental bacteria.

## Phage-Mediated Transduction

**FIGURE 1:**
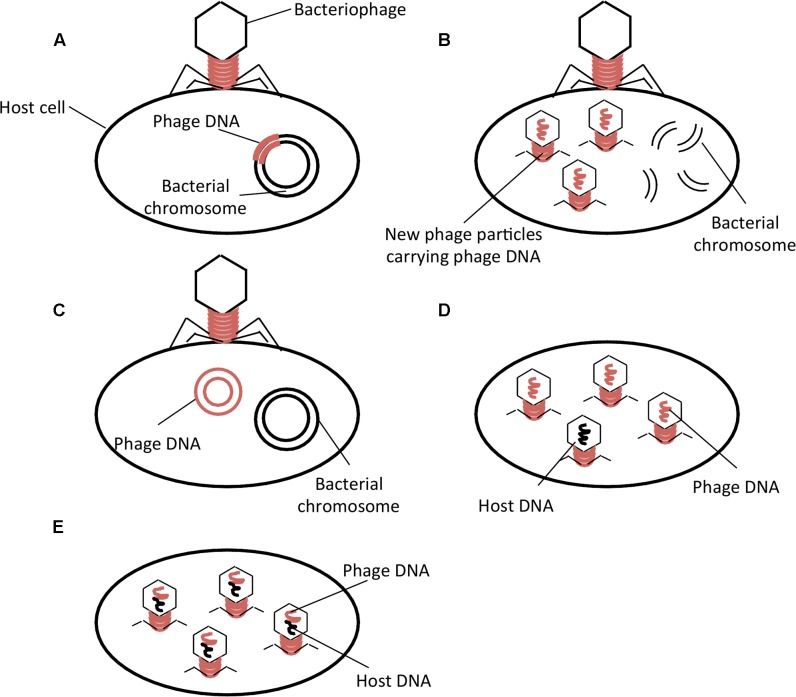
Phage life cycles and types of phage-mediated transduction. **(A)** Temperate phage life cycle; **(B)** Virulent phage life cycle; **(C)** Pseudolysogeny; **(D)** Generalized Transduction; **(E)** Specialized Transduction. Adapted from Feiner et al. (2015).

Transduction occurs by means of virulent and temperate phages (**Figures 1A–C**) (Suzuki and Griffiths, 2000; Feiner et al., 2015). Upon infection, temperate phages integrate their DNA into the host chromosome and the prophage may remain dormant in the host until some stress will induce the excision of the phage from the chromosome leading to subsequent formation of phage particles and lysis of the host cell (**Figure 1A**). Virulent phages do not integrate their DNA into the host chromosome but induce immediate formation of phage particles and lysis of the host cell (**Figure 1B**). Under unfavorable growth conditions, some phages can adopt a pseudolysogeny state where their genome does not degrade but instead exists as a plasmid within the cytoplasm and during bacterial cell division becomes incorporated into only one daughter cell (**Figure 1C**) (Feiner et al., 2015).

Two types of transduction, generalized and specialized, have been described. Generalized transduction refers to the mispackaging of bacterial DNA into the phage capsid (**Figure 1D**). The phage may then infect another susceptible host, thereby transferring genetic material to another bacterial cell where it will be integrated by homologous recombination. Specialized transduction is the improper excision of a prophage from the bacterial chromosome, which results in packaging of the bacterial DNA into phages at a higher frequency than generalized transduction (**Figure 1E**) (Griffiths, 2002). The lateral transfer of genes encoding antibiotic resistance by phage-mediated transduction could be an important contributing factor in the global spread of antibiotic resistance.

## Shiga Toxin-Producing *Escherichia coli*

Shiga toxin-producing *Escherichia coli* serotype O157:H7 was recognized as an important foodborne pathogen following two outbreaks that occurred in 1982 in the United States (Riley et al., 1983). Twenty-six cases were identified in Oregon, and 21 cases were reported in Michigan. The outbreaks were both linked to the consumption of hamburgers from a fast food restaurant chain, and *E. coli* O157:H7 was isolated from both patient stool samples and hamburger meat from the restaurant (Riley et al., 1983). Following the discovery of *E. coli* O157:H7, other serotypes of STEC have been identified, with many of these serotypes causing outbreaks and sporadic disease (Johnson et al., 2006). Numerous studies have detected the presence of antibiotic resistance genes in clinical and environmental STEC isolates. For example, resistance to streptomycin, sulfonamide, tetracycline, ampicillin, and cephalothin was found in isolates from human stool samples and in isolates procured from livestock and food (Kim et al., 1994; Schroeder et al., 2002). Multidrug resistant strains have also been observed with resistance to up to 10 different antimicrobials (Walsh et al., 2006).

Despite the non-specific nature of transduction, the phage mediated transfer of antibiotic resistance genes by transduction has been reported to occur in laboratory strains of *E. coli* (**Table 1**) (Tanyashin et al., 2003; Kenzaka et al., 2007; Marinus and Poteete, 2014). For instance, induction of Stx-converting generalized transducing phage 933W from *E. coli* 0157:H7 resulted in efficient transfer of tetracycline resistance genes to the *E. coli* laboratory strain, K-12 (Marinus and Poteete, 2014). In another study, Serra-Moreno et al. (2006) modified a Stx-converting bacteriophage, to incorporate genes encoding for resistance to tetracycline or chloramphenicol as markers for genetic transfer. Infection of *E. coli* DH5α and C600 with the recombinant phages resulted in production of transductants resistant to the respective antibiotics. In an attempt to explore the potential of phage mediated transduction as a vehicle of antibiotic resistance from food of animal origin to consumers, Shousha et al. (2015) tested retail chicken meat for the presence of coliphages capable of transducing antibiotic resistance genes. Of 243 coliphages, 24.7% were able to transduce one or more antibiotic genes, encoding for ampicillin, tetracycline, kanamycin and chloramphenicol, to the laboratory strain *E. coli* ATCC 13706 (Shousha et al., 2015). In another study, four phages isolated from wastewater effluent samples were observed to horizontally transfer a kanamycin resistance gene to strains of uropathogenic *E. coli* by generalized transduction (Battaglioli et al., 2011). Lastly, Kenzaka et al. (2007) developed a method termed CPRINS-FISH, to investigate the frequency of lateral gene transfer via transduction at the single-cell level, and demonstrated the transfer of the ampicillin resistance gene between *E. coli* cells at a surprisingly high frequency. Although phage-mediated horizontal transfer of antibiotic resistance genes between strains of STEC has not yet been directly shown, it is clear that Stx-phages can convert antibiotic susceptible *E. coli* strains to resistant strains *in vitro*. Therefore, it is reasonable to speculate that the transfer of antibiotic resistance genes via transduction occurs among Stx-producing *E. coli* serotypes, including O157:H7.

**Table 1:** Summary of phage-mediated transduction events demonstrated in *E. coli.*

Donor organism	Recipient organism	Phage	Antibiotic resistance gene	Reference
*E. coli* B834 (pBR322)	*E. coli* B_E_	Pseudo-T even phages	*Amp* and *tet* determinants of plasmid BR322	Tanyashin et al., 2003
*E. coli* HMS174 (pBR322)		RB42		
		RB43		
		RB49		

*E. coli* O157:H7 EDL933	*E. coli K12* AB1157	933W	*tet*	Marinus and Poteete, 2014

*E. coli* NBRC 12713 KEN1	*E. coli* NBRC 12713	P1*kc* T4GT7	*bla*	Kenzaka et al., 2007

*E. coli* NBRC 12713 KEN1	*E. coli* strains (C600 RK2, HB101, NBRC 12713, and W3110)	EC10	*bla*	Kenzaka et al., 2007

N/A	*E. coli* strains DH5α and C600	*Stx_2_* producing phages induced from STEC isolated from cattle: ØA9, ØA312, ØA534, ØA549, ØA557, ØVTB55 and 933W	*tet* and *cat*	Serra-Moreno et al., 2006

N/A	*E. coli* ATCC 13706	Sixty of 243 coliphages isolated from retail chicken meat could transduce antibiotic resistance genes	43 phages: ϕKmr	Shousha et al., 2015
			4 phages: ϕTetr	
			3 phages: ϕKmrAmpr	
			2 phages: ϕKmr Cmr	
			8 phages: ϕCmr	

N/A	*E. coli* CSH-2	P1*kc*	R factors:	Watanabe et al., 1968
			N-1: SU, SM, TC	
			N-3: TC, SM, TC	
			N-6: SU, SM, TC	
			N-9: TC	
			N-9: SM	
			R-15: SM, SU	
			S-a: CM, SM, CM	
			S-b: SU, SM, CM, TC	
			S-b: TC	
			K: SU, SM, CM, TC	
			K-R_3_: SU, SM, CM	
			K-TC: TC	

*E. coli* CFT073 WAM3686	*E. coli* CFT073 WAM2909	Four phages isolated from wastewater effluents: ΦEB49, ΦEB47 ΦEB32, ΦEB5	*kan*	Battaglioli et al., 2011

**amp:* ampicillin; *tet,* TC: tetracycline; *bla*: beta-lactamase; *kan,*ϕKmr: kanamycin; *cat*: chloramphenicol acetyl transferase gene; ϕCmr, CM: chloramphenicol; ϕKmr Ampr: kanamycin and ampicillin; ϕKmr Cmr: kanamycin and chloramphenicol; SU: sulfonamide; SM: streptomycin.*

There have also been reports linking antibiotic resistance to specific phage types, suggesting a phage-mediated bias toward antibiotic resistance (Mora et al., 2005; Ziebell et al., 2008; Ziebell et al., 2011). In a Canadian study, Ziebell et al. (2011) characterized 187 isolates of STEC O157:H7 and observed that 45 of these possessed resistance to streptomycin, sulfonamide, and tetracycline. Of the 45 isolates, 43 belonged to three phage types: PT23, PT45, and PT67. Another study using isolates from Spain also recorded an association between phage type and resistance to the same three antibiotics (Mora et al., 2005). However, phage types found to be linked to antibiotic resistance were different from the Canadian study and included PT21/28, PT23, PT34, and PT2. The proposed link between phage type and antibiotic resistance could suggest a potential role for phages in the dissemination of antibiotic resistance. While Ziebell et al. (2008) believed the resistance genes they studied were stably incorporated into the bacterial genome before the bacterium differentiated into different phage types, it is also possible that certain phage types are prone to acquiring and transferring select resistance genes.

Another instance of phage-mediated transduction of antibiotic resistance in *E. coli,* stems from the debated use of antimicrobials as growth promoters in food-producing animals (Economou and Gousia, 2015). Carbadox, marketed under the name MECADOX, is an agricultural antimicrobial added to swine feed in the United States to prevent swine dysentery, improve feed efficiency and promote weight gain and growth in swine (Bearson et al., 2014; Food and Drug Administration [FDA], 2015). A laboratory-based study established that Stx-converting prophages were induced by various concentrations of carbadox (0.5–8 ppms) from an *E. coli* C600 strain containing Stx-phage 933W and three other clinical Shiga toxin-producing *E. coli* strains (Köhler et al., 2000). The concentrations of carbadox identified to be sufficient for induction were approximately 0.5–100 times lower than that found in the gut content isolated from the intestine of carbadox-fed piglets (Graaf et al., 1988; Köhler et al., 2000). These findings raise concern because, as previously discussed, the Stx-prophage 933W has the ability to transfer tetracycline resistant cassettes by generalized transduction, at least in a laboratory setting (Marinus and Poteete, 2014). Moreover, olaquindox, another agricultural antimicrobial permitted in China, was three times more capable of inducing phage 933W than carbadox (Köhler et al., 2000). Although these results were observed in the laboratory, carbadox could potentially induce prophages from *E. coli* strains in the environment. For example, Allen et al. (2011) demonstrated that carbadox was capable of inducing more prophages in bacterial strains isolated from the gut of carbadox-treated swine compared to that of untreated swine. The implications of phage transfer of antibiotic resistance genes in the food animal environment are clear. For example, once prophages, harbored within *E. coli*, are released, they could transfer antibiotic resistance genes to foodborne pathogens such as *E. coli* O157:H7 or *Salmonella* spp. within the gastrointestinal tract of cattle or swine. These now antibiotic resistant foodborne pathogens would be present in the stool of cattle or swine, potentially leading to environmental contamination of crops, thereby contributing to fresh produce outbreaks (Jung et al., 2014). On the other hand, during slaughter, resistant foodborne pathogens could contaminate meat products destined for human consumption, putting consumers at risk of antibiotic resistant infections.

Conversely, although studies appear to indicate that transduction of antibiotic resistance genes does occur, there is some uncertainty as to how much transduction contributes to overall lateral gene transfer. Using conventional plating methods, the estimates of the frequency of phage-mediated DNA transfer ranged between 10^-9^ and 10^-5^ per bacteriophage (Weinbauer and Rassoulzadegan, 2004; Kenzaka et al., 2007). However, using the CPRINS-FISH method, Kenzaka et al. (2007) showed that gene transfer by phages in *E. coli* cells occurs at a much higher frequency (ranging between 10^-4^ and 10^-3^ for the phages tested) than previously thought. They attributed the previous underestimation to the limitations in traditional culturing and enumeration methods. However, even in the best conditions, modeling of infection dynamics shows that the contribution of generalized transduction to lateral gene spread is small (Volkova et al., 2014). The model developed by Volkova et al. (2014) accounts for many factors that may contribute to transduction such as phage dynamics, lysogens, host range, and bacterial susceptibility to the antibiotic resistance gene of interest. This model was constructed for temperate phages infecting *E. coli* within the lumen of the bovine large intestine. However, virulent phages may play a role in transduction of antibiotic resistance as well. Furthermore, only phages infecting *E. coli* specifically were considered when developing the model, and though it may be an indicator, bacteria and phages are highly diverse. Hence, it is difficult to evaluate if predictions derived from the model are applicable across species and types. Finally, the model is restricted to the bovine large intestine and estimates the average *E. coli* residency time in the intestine to be on the order of 100 h. Though the presence of *E. coli* makes the intestine an optimal setting for transduction to occur, transduction is not limited to this location, and the possibility of phage mediated spread of antibiotic resistance genes occurring in the environment cannot be discounted. There are also exterior factors, such as the use of antibiotics that may induce excision of prophages. The number of factors that may, or may not, influence transduction and gene transfer is large, and therefore, transduction as a means of lateral gene transfer in *E. coli* should not be quickly dismissed.

## Salmonella enterica

Multidrug resistance amongst *Salmonella* spp. have globally increased during the past two decades. For instance, in Great Britain, between 1994 and 2010, *Salmonella* Typhimurium was the most common serovar isolated from swine (Mueller-Doblies et al., 2013). When tested against a panel of antimicrobials, its resistance to six or more antimicrobials increased from 27.2% in 1994 to 58.3% in 2010. In Spain, antimicrobial resistance of different *Salmonella* serovars isolated from retail poultry increased from resistance to 3.98 antimicrobials in 1993 to 5 antimicrobials in 2006 (Alvarez-Fernandez et al., 2012). In China, 80% of *Salmonella* isolates from retail meats were resistant to one antimicrobial and 53% were resistant to more than three antimicrobials, with *S.* Enteritidis being the predominant serovar (Yang et al., 2010).

The prevalence of penta-resistant strains of *S.* Typhimurium phage type DT104 has been increasing exponentially since its emergence in 1989, particularly in Europe and North America (Threlfall, 2000; Helms et al., 2005). In phage type DT104, the penta-resistance genes are clustered on a 43-kb SGI1, which is flanked by two type I integrons (Boyd et al., 2002). Horizontal transfer of the penta-resistance is hypothesized to be facilitated by two P22-like prophages, ST104 or PDT17, harbored within DT104 (Schmieger and Schicklmaier, 1999; Tanaka et al., 2004). P22 is a well-known *Salmonella* phage extensively used in molecular biology for its ability to introduce foreign genes by generalized and specialized transduction (Zinder and Lederberg, 1952; Smith and Levine, 1965; Smith and Stocker, 1966; Smith-Keary, 1966; Wing, 1968). Schmieger and Schicklmaier (1999), determined that when P22-like phages, ES18 and PDT17, were released from DT104, they could transduce antibiotic resistance genes (**Table 2**). The researchers demonstrated the transduction of *cam* and *amp* by phage PDT17 and *amp*, *cam,* and *tet*, which confer resistance to ampicillin, chloramphenicol, and tetracycline, respectively, by ES18 from a donor DT104 strain into a DT104 recipient strain lacking these resistance genes. Also detected was the co-transduction of selected resistance genes by phage ES18. Most notably, of 145 *cam* transductants, all but one co-transduced *amp* and *tet*, and of 71 *tet* transductants, all but one co-transduced *amp* and *cam.* Furthermore, in 14 of 16 transductants, Schmieger and Schicklmaier (1999) observed that ES18 could co-transduce *sul* and *str*, genes involved in resistance to sulfonamides and streptomycin, respectively, together with *amp*, *cam,* and *tet* to create the ACSSuT resistance phenotype (). This co-transduction most likely occurs because *amp* and *str* are located on the integrons flanking SGI1, and the phage likely packages the SGI1 and its flanking integrons (Ridley and Threlfall, 1998; Sandvang et al., 1998; Mulvey et al., 2006). In another study, Bearson et al. (2014) investigated whether a mixture of phages induced from *S.* Typhimurium DT104 by carbadox, an agricultural antimicrobial discussed above, could transduce SGI1 to a DT104 strain with sensitivity to ampicillin, chloramphenicol, and tetracycline due to an internal deletion within SGI1. The authors observed the transduction of tetracycline resistance and 100% co-transduction of *floR* encoding for chloramphenicol and *bla*_pse-1_ encoding for ampicillin, as seen by Schmieger and Schicklmaier (1999). These authors suggested that the carbadox induced phage transduction in *S.* Typhimurium DT104 and another ACSSuT resistant strain, DT120, is common after observing frequent transduction events of the *his* operon. Phage P22 was detected within several DT104 and DT120 isolates shown to be capable of generalized transduction. The absence of transduction following carbadox induction of a DT104 strain with a deletion of a P22-like prophage suggests that P22-like prophages are responsible for generalized transduction in DT104. This was further supported by the incapability of transduction in four *S.* Typhimurium strains that do not contain P22-like phages. These studies provide evidence that transduction and co-transduction by P22-like prophages of antibiotic resistance genes co-located within SGI1 in multidrug-resistant *S.* Typhimurium strains, is a common phenomenon. Moreover, genome scanning demonstrated that P22-like prophages are common in 18 *Salmonella* serovars suggesting that generalized transduction may be underestimated (Bearson et al., 2014).

**Table 2:** Summary of phage-mediated transduction events demonstrated in *Salmonella* and the agricultural soil microbiome.

Donor organism	Recipient organism	Phage	Antibiotic resistance gene	Reference
*S.* Typhimurium DT104 strain DT17	*S.* Typhimurium DT104 strain DT16	ES18	PDT17: *amp, cam*	Schmieger and Schicklmaier, 1999
Resistance profile ACS(sp)SuT				
	Resistance profile			
	SSu			
		PDT17		
			ES18 co-transduction:	
			*Amp cam*	
			*Sul str*	

*S.* Typhimurium DT104 NCTC13348	*S.* Typhimurium DT104 strain	Phages induced by carbadox from donor organism	Transduction: *tetG*	Bearson et al., 2014
	BBS 1012			
	Internal deletion in SI-1 for ampicillin, chloramphenicol, and tetracycline			
			Co-transduction:	
			*floR*	
			*bla*_pse-1_	

N/A	*S.* Typhimurium LT2	P22	R factors:	Watanabe et al., 1968
			N-1: TC	
			N-3: TC, SU, SM	
			N-3: SU, SM, TC	
			N-3: SU, SM	
			N-6: SU, SM, TC	
			N-9: TC	
			N-9: TC, SU, SM	
			S-a: CM, SU, SM	
			K: TC	
			K-TC: TC	

*S.* Heidelberg S25	*S.* Typhimurium MZ1262	P24	*bla*_CMY -2_	Zhang and Lejeune, 2008
			*tet*(A)	
			*tet*(B)	

*S.* Typhimurium DT120 (BBS 1162)	*S.* Typhimurium BBS 243	Fluoroquinolone induced phages from donor organisms	Plasmid conferring kanamycin resistance native to donor organisms	Bearson and Brunelle, 2015
*S.* Typhimurium DT104 (BBS 1170)				

N/A	Soil coliforms	Isolated and enriched biosolid phages	Cefoxitin Sulfamethazine	Ross and Topp, 2015

**amp,* bla_*pse-1*_*:* ampicillin; *tet,*ϕTetr, TC: tetracycline; *cam, floR,*ϕCmr, CM*:* chloramphenicol; *kan,*ϕKmr: kanamycin; *sul,* SU: sulfonamide; *str,* SM*:* streptomycin; *bla*_*CMY-2*_: beta-lactamase; ϕKmr Ampr: kanamycin and ampicillin; ϕKmr Cmr: kanamycin and chloramphenicol*.

R factors may represent another form of MGE that can be horizontally transferred via phage-mediated transduction. An R factor is an episome that harbors one or more antibiotic resistance determinants. R factors were first discovered in Japan in the 1940s when resistance emerged to sulfonamides, which were being used to treat dysentery caused by *Shigella* spp. (Mitsuhashi, 1993). Shortly after, streptomycin, chloramphenicol, and tetracycline became the treatments of choice but resistance to these antibiotics soon emerged as a result of an R factor harbored within *Shigella* strains (Ochiai et al., 1959; Akiba et al., 1960; Nakaya et al., 1960). There have been numerous studies demonstrating that conjugative R factors spread antibiotic resistance genes between *Salmonella* strains, as well as *E. coli* strains (Ishiguro et al., 1980; Balis et al., 1996; Lázaro et al., 2004), and one study reported that the transfer occurred by transduction in *Salmonella* as well as *E. coli* (Watanabe et al., 1968). In this study, Watanabe et al. (1968) isolated various R factors from *Shigella* strains with different MIC’s of resistance to sulfanilamide, streptomycin, chloramphenicol, and tetracycline, and demonstrated successful transduction of the R factors by phage P22 to *S.* Typhimurium LT-2, as well as to *E. coli* CSH-2 by phage P1. Following transduction of the R factors, the group attempted to transfer the R factor by conjugation. Many of the transductants were unable to transmit their drug resistance genes suggesting that the R factor had integrated into the host’s chromosome (Watanabe et al., 1968). The P22-like phages epsilon34, ES18, P22, ST104, and ST64T are all inducible and frequently lysogenize *Salmonella* (Kropinski et al., 2007). The integration of R factors into or in close proximity to P22-like prophages would facilitate the packaging of the R factor into the head of the assembling phage during induction from its host, thus contributing to the spread of antibiotic resistance within bacteria capable of causing foodborne illnesses, in the intestinal flora of livestock and in the environment.

Another aspect that is of concern is the worldwide increase in resistance to third-generation cephalosporins, which are used to treat invasive *Salmonella* infection (Eller et al., 2013; Seiffert et al., 2013; Noda et al., 2015; Liakopoulos et al., 2016). Resistance to cephalosporins is mainly due to ESBLs, such as TEM-, SHV-, and CTX-M, or plasmid mediated AmpC β-lactamases (pAmpCs), such as CMY, encoded on transferrable conjugative plasmids (Carattoli et al., 2002; Guerra et al., 2002; Chen et al., 2004). Still, Zhang and Lejeune (2008) demonstrated that they could also be transferred by generalized transduction. In this work, the authors observed that phage P24, induced from an isolate of *S.* Typhimurium, was capable of propagating on a multidrug resistant strain, *S.* Heidelberg (S25). Therefore, S25 harboring phage P24 was used as transduction donor to transfer ESBL and tetracycline resistance genes to a recipient *S.* Typhimurium isolate. PCR confirmed the presence of *bla*_CMY -2_, *tet*(A), and *tet*(B) in various *S.* Typhimurium transductants. Tetracycline genes were not co-transduced with *bla*_CMY -2_, however, their transduction frequency was equivalent, indicating generalized transduction. This study is the first report of antibiotic resistance genes transferred by phage-mediated transduction between different *Salmonella* serovars. Although this may be the first documented report, it is likely that cross-serovar transduction occurs frequently because phages can bind to various surface protein receptors on different species and serovars. The LPS, FliC, OmpC, OmpF, OmpA, are examples of phage receptors present in STEC and *Salmonella* (Rakhuba et al., 2010). As further evidence, Zhang and Lejeune (2008) observed that 13 inducible phages recovered from 31 *Salmonella* serovars were capable of propagating on two or more *Salmonella* serovars including those often responsible for outbreaks such as Heidelberg, Enteritidis, and Typhimurium as well as Kentucky, which is one of the most common serovars detected in chickens and ground chicken meat in Europe. This study demonstrates that phage-mediated transduction can contribute to the spread of antibiotic resistance in various *Salmonella* serovars associated with foodborne outbreaks.

The other antibiotic of choice for treatment of invasive *Salmonella* infections is ciprofloxacin, a fluoroquinolone (Eng et al., 2015). Ciprofloxacin resistance has dramatically increased in food and clinical isolates (Lin et al., 2015). Its resistance in *Salmonella* is mainly attributed to double mutations in *gyrA* and a single mutation in *parC.* In addition, it’s suggested that *oqxAB* operon is responsible for an increase in resistant clinical *Salmonella* strains (Wong et al., 2014). Bearson and Brunelle (2015) sought to investigate whether exposure to ciprofloxacin, as well as fluoroquinolones enrofloxacin and danofloxacin, which are used in veterinary medicine, would induce phage-mediated transfer of a native plasmid encoding kanamycin resistance by generalized transduction from *Salmonella* DT104 and DT102. The authors observed that all three fluoroquinolones stimulated generalized transduction of the kanamycin resistance plasmid to a strain of *Salmonella* Typhimurium. Since *Salmonella* frequently colonize cattle, swine and poultry without causing clinical disease, the authors expressed concern that there is potential for a scenario in which fluoroquinolones are administered to animals that are asymptomatically colonized. This would induce phage-mediated generalized transduction and increase the horizontal transfer of antibiotic resistance genes. Moreover, the administered fluoroquinolones would be excreted in the feces and urine of these food producing animals and stimulate horizontal gene transfer in the environment (Bearson and Brunelle, 2015).

As seen by the frequency of multi-drug resistance patterns, the transfer of resistance genes occurs extensively in strains of foodborne *Salmonella*, and transduction appears to play a prominent role in the successful dissemination of antibiotic resistance among the *Salmonella*e.

## Environmental Factors Associated with Bacteriophage Transduction of Antibiotic Resistance Genes

**FIGURE 2:**
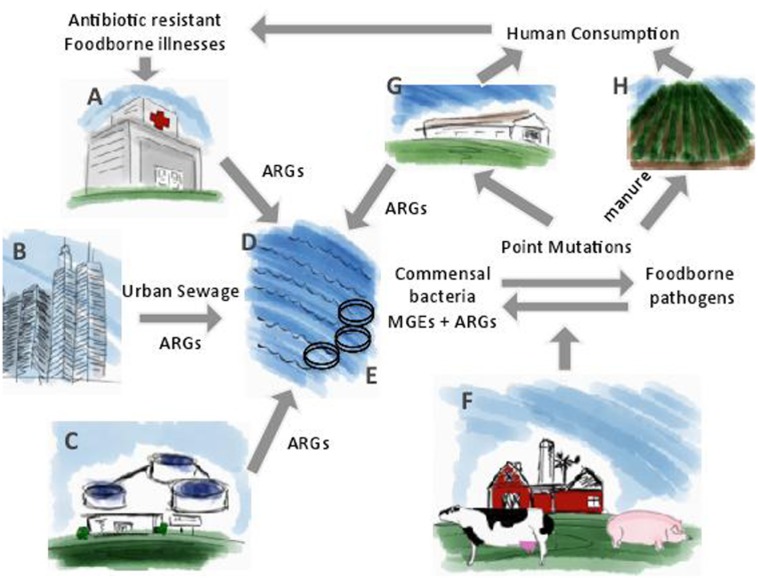
The cycle for the dissemination of antibiotic resistance genes from the environment to human consumption. **(A)** Hospital effluents; **(B)** Urban sewage; **(C)** Wastewater treatment plant effluents; **(D)** Bodies of water such as lakes and oceans; **(E)** Aquaculture; **(F)** Food-producing animals; **(G)** Slaughterhouse; **(H)** Manure application. Adapted from Muniesa et al. (2013).

A number of factors contribute to the dissemination of antibiotic resistance such as the use and misuse of antibiotics in medicine, agriculture, and aquaculture. The emergence and persistence of antibiotic resistant bacteria impacts the environment through wastewater treatment plants, farm and slaughterhouse runoffs, hospital effluents, manure applications, and aquaculture. Muniesa et al. (2013) proposed a model to explain the mobilization of antibiotic resistance genes by phages in the environment. These researchers proposed that phages harboring antibiotic resistance genes present in different environmental biomes, are mobilized to commensal bacteria of animal and human biomes. From commensal bacteria, antibiotic resistance genes are transferred to pathogens of the *Enterobacteriaceae* family, such as *Salmonella* and *E. coli* O157:H7. Under selective pressure exerted by antibiotics, antibiotic resistance genes are incorporated into MGEs and will continue their mobilization through the animal biomes and human biomes by horizontal gene transfer. Variations of antibiotic resistance genes incorporated on MGEs will also develop through point mutations (Muniesa et al., 2013). In addition to this model, antibiotic resistant foodborne pathogens can be transferred from livestock to humans through improperly cooked meat and cross-contamination (Centers for Disease Control and Prevention [CDC], 2002; Schneider et al., 2011). Irrigation water and manure application contaminate crops, which also leads to antibiotic resistant foodborne illnesses. Finally, antibiotic resistance genes return to the environment through urban sewage and wastewater effluents and the cycle begins again (**Figure 2**). If left unchecked, this cycle has the potential to greatly compromise the effectiveness of antibiotic therapeutics.

Evidence of phage-mediated transduction events has been investigated in different environmental niches harboring phages and in different antibiotic resistant bacteria. For example, Colomer-Lluch et al. (2011) investigated whether two prominent β-lactamase resistance genes, *bla*_TEM_ and *bla*_CTX-M_, were present in phages collected from urban sewage or in the Llobregat river, near Barcelona, Spain. The sewage plant in Barcelona services approximately 500,000 inhabitants, and the river contains human and animal runoffs. The authors detected 10^2^–10^4^ gene copies of *bla*_TEM_ in DNA of phages isolated from urban sewage. Phages from the river samples contained, on average, 10 less gene copies of *bla*_TEM_ than phages isolated from sewage. Presence of *bla*_CTX-M_ was similar to that of *bla*_TEM_; 1.5 to 3 log_10_ units were detected in phages isolated from urban sewage samples and less than 1 log_10_ unit was detected in phages from the river samples. The authors then evaluated the viability of these resistance genes by examining whether they could confer resistance to the bacteria when transfected into two *E. coli* recipients. They detected *bla*_TEM_ in a greater percentage than *bla*_CTX-M_ in both strains. The authors suggested that in the presence of susceptible recipient strains, these environmental conditions would facilitate the phage-mediated transduction of ESBL’s in the environment. As further evidence, Marti et al. (2014) demonstrated that phages carrying antibiotic resistance genes persist longer in the environment than their bacterial hosts. These authors investigated the presence of ESBLs (*bla*_TEM_, *bla*_CTX-M_, and *bla*_SHV_) and fluoroquinolone resistance genes (*qnrA, qnrB,* and *qnrS)* from two wastewater treatment plants and two hospitals located in Catalonia, Spain. A decrease in antibiotic resistance genes was observed in bacterial DNA from wastewater treatment plant samples compared to hospitals samples, which is unsurprising since wastewater treatment decreases the microbial load. However, antibiotic resistance genes, *qnrS* and *bla*_SHV_ were detected in the phage DNA of all samples providing evidence of the persistence of phages and their potential to act as vectors for horizontal transfer of antibiotic resistance genes in the environment.

A study in Spain quantified quinolone resistance genes, *qnrA* and *qnrS,* in DNA of phage particles isolated from raw urban wastewater, cattle and poultry slaughterhouse wastewater and from the Llobregat river near Barcelona, Spain (Colomer-Lluch et al., 2014). Q*nrA* was most prevalent because it was identified in the phage DNA of 100% of urban waste and river samples and 71.4% of animal wastewater samples. To evaluate the influence of phage inducing factors on the dissemination of antibiotic resistance genes (i.e., *qnrA, qnrS*, *bla*_TEM_, and *bla*_CTX_) in the environment, mitomycin C, ciprofloxacin, EDTA, and sodium citrate were used to induce phages from wastewater samples. Surprisingly, these authors observed that mitomycin C and ciprofloxacin, two known inducers of temperate phages, had no effect on the number of phages carrying *qnr*. However, EDTA and sodium citrate demonstrated a significant increase in phages carrying *qnrA, qnrS*, *bla*_TEM_, and *bla*_CTX_ (Colomer-Lluch et al., 2014). This is probably attributed to the chelating properties of EDTA and sodium citrate, which disrupt the outer bacterial membrane by chelating the magnesium present within the LPS and cause induction of the phages (Imamovic and Muniesa, 2012). Based on these results, Colomer-Lluch et al. (2014) hypothesized that these phage particles carrying antibiotic resistance genes are generalized transducing phages since they contain fragments of bacterial DNA and no phage DNA. Thus, the phage particles lack the necessary genes for phage growth and lysis making them unable to produce plaques. Furthermore, to further support their hypothesis, they argued that no increase in phages carrying *qnr* genes was observed when induced with mitomycin C and ciprofloxacin, which are generally effective against specialized transducing phages. Additionally, the authors argued that since they did not observe an increase in virulent phages during induction, this further supports the hypothesis that the mobilization of *qnrA, qnrS*, *bla*_TEM_, and *bla*_CTX_ was due to generalized transducing phages. This study provides evidence that the presence of phage-encoded antibiotic resistance genes in slaughterhouse run offs and river samples can be hotspots for generalized transduction contributing to the emergence of new resistance strains.

Agricultural soils have also been investigated as a reservoir for phages, which carry antibiotic resistance genes, as well as the potential of such phages for horizontal gene transfer in the agricultural soil microbiome. Ross and Topp (2015), assessed the abundance of antibiotic resistance genes within the bacterial fraction and phage fraction of soil mixed with raw, digested, dewatered, and composted manure prior to application, in contrast to soil without the application of manure. Antibiotic resistance genes were more abundant in bacterial fractions of soil applied with raw, digested and dewatered manure compared to composted manure, which indicated the efficiency of this practice in reducing the presence of antibiotic resistance gene carrying bacteria. However, the abundance of antibiotic resistance genes remained steady in the phage fraction of all manure applications. In addition, the reservoir of antibiotic resistance genes in the phage fraction remained constant with the application of treated biosolids, defined as nutrient rich organic materials produced from treated sewage sludge and used as a fertilizer in agriculture. Taken together, these results suggested that soilborne phages are a reservoir of antibiotic resistance genes. Moreover, the authors investigated the potential for transduction of antibiotic resistance genes by phages isolated from biosolids to soil coliforms, when selective pressure (1/10 and 1/100 of the breakpoint concentration of cefoxitin and sulfamethazine antibiotics, respectively), was applied. A 3.7- and 7.1-fold increase of cefoxitin-resistant coliforms and sulfamethazine-resistant coliforms, respectively, was observed (**Table 2**). This study provides evidence that regardless of the type of manure treatment, the soil is a reservoir of antibiotic resistance genes due to the presence of soilborne phages and antibiotics represent a form of selective pressure that promotes the horizontal transfer of antibiotic resistance genes by transduction (Ross and Topp, 2015).

## Phages May Not Play a Large Role in the Spread of Antimicrobial Resistance Genes Among Bacteria

Reasonable evidence exists supporting the concept that phage-mediated transduction is a contributing factor to the dissemination of antibiotic resistance in foodborne pathogens *E. coli* and *Salmonella* spp. Still, the role that phages play in transduction of antibiotic resistance genes remains controversial, as evidenced by two studies highlighted here. In the first study, Enault et al. (2017) employed several bioinformatic approaches to identify and assess the presence of antibiotic resistance genes within phage genomes, and found that antibiotic resistance gene prevalence in 1,181 phage genomes were vastly over-estimated due to low homologies with antibiotic resistance genes, as well as matches to proteins unrelated to antibiotic resistance. Furthermore, re-analysis of human- or mouse-associated viromes for antibiotic resistance genes suggested that the presence of such genes as attributable to phages in viromes were previously over-estimated. The authors concluded that their findings show that antibiotic resistance genes are rarely encoded in phages.

In the second study, Allen et al. (2011) directly studied prophage induction and horizontal gene transfer in animals. In this work, the authors used metagenomics to evaluate the effect of two antibiotics in feed (carbadox and ASP250 [chlortetracycline, sulfamethazine, and penicillin]) on swine intestinal phage metagenomes (viromes). They also monitored the bacterial communities using 16S rRNA gene sequencing. It was observed that ASP250, but not carbadox, caused significant population shifts in both the phage and bacterial communities. Of importance to the topic of phage transduction, the authors observed that antibiotic resistance genes, such as multidrug resistance efflux pumps, were identified in the viromes, but in-feed antibiotics caused no significant changes in their abundance. The abundance of phage integrase-encoding genes was significantly increased in the viromes of medicated swine over that in the viromes of non-medicated swine, demonstrating the induction of prophages with antibiotic treatment. This suggests that while prophages were induced in the swine gut, this did not result in horizontal gene transfer of ARGs from the prophages to bacteria in the swine gut.

## Conclusion

Notwithstanding these studies, and given the ubiquity, abundance and resilience of phages and the role of the environment as a reservoir, the contribution of transduction to the spread of antibiotic resistance must be considered and should be further explored in future studies. Furthermore, reasonable evidence supports the concept that phage-mediated transduction is a contributing factor to the dissemination of antibiotic resistance in *E. coli* O157:H7 and NTS. Controlling the spread of antibiotic resistance is an ongoing concern that must be addressed in order to ensure that a primary defense against infectious disease remains effective. Future studies should also address methods by which phage-based spread of antibiotic resistance genes would be mitigated. On this note, several studies have shown that phage-transferable CRISPR-Cas systems are capable of killing pathogens and resensitizing them to antibiotics (Bikard et al., 2014; Citorik et al., 2014). A recent study by Yosef et al. (2015) is noteworthy. In this work, the authors used phages to deliver a CRISPR-associated (Cas) to reverse antibiotic resistance and eliminate the transfer of resistance between strains. Instead of directly killing bacterial pathogens, as in traditional phage therapy, the bacteria were instead resensitized to the antibiotics that they are resistant to. While this work represented a proof of concept, as only bacterial isolates susceptible to the genetically modified phage would be resensitized, this study nevertheless demonstrates the potential for antibiotic resistance to be reversed through the use of novel approaches.

## Author Contributions

AC, BC, AL, and LG drafted and edited the manuscript. All authors revised the manuscript.

## Conflict of Interest Statement

The authors declare that the research was conducted in the absence of any commercial or financial relationships that could be construed as a potential conflict of interest.

## Abbreviations

ACSSuT: penta-resistance to ampicillin, chloramphenicol, streptomycin, sulfonamides, and tetracycline
ARG: antibiotic resistant gene
CPRINS-FISH: cycling primed *in situ* amplification-fluorescent *in situ* hybridization
ESBL: extended-spectrum β-lactamase
MGE: mobile genetic element
NTS: non-typhoidal *Salmonella*
PBP: penicillin binding protein
SGI1: *Salmonella* genomic island 1
Stx: Shiga toxin
TMP-SMZ: trimethoprim-sulfamethoxazole.

## References

[B1] Acheson D. Hohmann E. L. (2001). Nontyphoidal salmonellosis. *Clin. Infect. Dis.* 32 263–269. 10.1086/318457 11170916

[B2] Akiba T. Koyama K. Ishiki Y. Kimura S. Fukushima T. (1960). On the mechanism of the development of multiple-drug-resistant clones of *Shigella*. *Jpn. J. Microbiol.* 4 219–227. 10.1111/j.1348-0421.1960.tb00170.x 13681921

[B3] Allen H. K. Looft T. Bayles D. O. Humphrey S. Levine U. Y. Alt D. (2011). Antibiotics in feed induce prophages in swine fecal microbiomes. *mBio* 2:e260-11. 10.1128/mBio.00260-11 PMC3225969 22128350

[B4] Alvarez-Fernandez E. Alonso-Calleja C. Garcia-Fernandez C. Capita R. (2012). Prevalence and antimicrobial resistance of *Salmonella* serotypes isolated from poultry in Spain: comparison between 1993 and 2006. *Int. J. Food Microbiol.* 153 281–287. 10.1016/j.ijfoodmicro.2011.11.011 22208955

[B5] Balis E. Vatopoulos A. C. Kanelopoulou M. Mainas E. Hatzoudis G. Kontogianni V. (1996). Indications of in vivo transfer of an epidemic R plasmid from *Salmonella* enteritidis to *Escherichia coli* of the normal human gut flora. *J. Clin. Microbiol.* 34 977–979. 8815122 10.1128/jcm.34.4.977-979.1996 PMC228931

[B6] Battaglioli E. Baisa G. Weeks A. Schroll R. Hryckowian A. Welch R. (2011). Isolation of generalized transducing bacteriophages for uropathogenic strains of *Escherichia coli*. *Appl. Environ. Microbiol.* 77 6630–6635. 10.1128/AEM.05307-11 21784916 PMC3187168

[B7] Bearson B. L. Allen H. K. Brunelle B. W. Lee I. S. Casjens S. R. Stanton T. B. (2014). The agricultural antibiotic carbadox induces phage-mediated gene transfer in *Salmonella*. *Front Microbiol.* 5:52 10.3389/fmicb.2014.00052 PMC3920066 24575089

[B8] Bearson B. L. Brunelle B. W. (2015). Fluoroquinolone induction of phage-mediated gene transfer in multidrug-resistant *Salmonella*. *Int. J. Antimicrob. Agents* 46 201–204. 10.1016/j.ijantimicag.2015.04.008 26078016

[B9] Bielaszewska M. Idelevich E. A. Zhang W. Bauwens A. Schaumburg F. Mellmann A. (2012). Effects of antibiotics on Shiga toxin 2 production and bacteriophage induction by epidemic *Escherichia coli* O104:H4 strain. *Antimicrob. Agents Chemother.* 56 3277–3282. 10.1128/AAC.06315-11 22391549 PMC3370775

[B10] Bikard D. Euler C. W. Jiang W. Nussenzweig P. M. Goldberg G. W. Duportet X. (2014). Exploiting CRISPR-Cas nucleases to produce sequence-specific antimicrobials. *Nat. Biotechnol.* 32 1146–1150. 10.1038/nbt.3043 25282355 PMC4317352

[B11] Boyd D. Cloeckaert A. Chaslus-Dancla E. Mulvey M. R. (2002). Characterization of variant *Salmonella* genomic island 1 multidrug resistance regions from serovars Typhimurium DT104 and Agona. *Antimicrob. Agents Chemother.* 46 1714–1722. 10.1128/AAC.46.6.1714-1722.2002 12019080 PMC127246

[B12] Carattoli A. Tosini F. Giles W. P. Rupp M. E. Hinrichs S. H. Angulo F. J. (2002). Characterization of plasmids carrying CMY-2 from expanded-spectrum cephalosporin-resistant *Salmonella* strains isolated in the United States between 1996 and 1998. *Antimicrob. Agents Chemother.* 46 1269–1272. 10.1128/AAC.46.5.1269-1272.2002 11959555 PMC127137

[B13] Centers for Disease Control and Prevention [CDC] (2002). *Outbreak of Multidrug-Resistant Salmonella Newport–United States, January-April 2002*. Available at: http://www.cdc.gov/mmwr/preview/mmwrhtml/mm5125a1.htm 12118534

[B14] Centers for Disease Control and Prevention [CDC] (2015a). *Antibiotic Resistance in Foodborne Germs is an Ongoing Threat.* Available at: https://www.cdc.gov/media/releases/2015/a0609-antibiotic-resistance.html [accessed May 9 2017].

[B15] Centers for Disease Control and Prevention [CDC] (2015b). *Salmonella Enteritidis Infections Linked to Sprouts.* Available at: https://www.cdc.gov/Salmonella/enteritidis-11-14/ [accessed May 9 2017].

[B16] Chen S. Zhao S. White D. G. Schroeder C. M. Lu R. Yang H. (2004). Characterization of multiple-antimicrobial-resistant *Salmonella* serovars isolated from retail meats. *Appl. Environ. Microbiol.* 70 1–7. 10.1128/AEM.70.1.1-7.2004 14711619 PMC321239

[B17] Citorik R. J. Mimee M. Lu T. K. (2014). Sequence-specific antimicrobials using efficiently delivered RNA-guided nucleases. *Nat. Biotechnol.* 32 1141–1145. 10.1038/nbt.3011 25240928 PMC4237163

[B18] Clokie M. R. Millard A. D. Letarov A. V. Heaphy S. (2011). Phages in nature. *Bacteriophage* 1 31–45. 10.4161/bact.1.1.14942 21687533 PMC3109452

[B19] Colomer-Lluch M. Jofre J. Muniesa M. (2011). Antibiotic resistance genes in the bacteriophage DNA fraction of environmental samples. *PLoS ONE* 6:e17549 10.1371/journal.pone.0017549 PMC3048399 21390233

[B20] Colomer-Lluch M. Jofre J. Muniesa M. (2014). Quinolone resistance genes (qnrA and qnrS) in bacteriophage particles from wastewater samples and the effect of inducing agents on packaged antibiotic resistance genes. *J. Antimicrob. Chemother.* 69 1265–1274. 10.1093/jac/dkt528 24458509

[B21] Dewaal C. S. Grooters S. (2013). *Antibiotic Resistance in Foodborne Pathogens.* Washington, DC: Center for Science in the Public Interest 1–22.

[B22] Economou V. Gousia P. (2015). Agriculture and food animals as a source of antimicrobial-resistant bacteria. *Infect. Drug Resist.* 8 49–61. 10.2147/IDR.S55778 25878509 PMC4388096

[B23] Eller C. Simon S. Miller T. Frick J.-S. Prager R. Rabsch W. (2013). Presence of β-lactamases in extended-spectrum-cephalosporin-resistant *Salmonella enterica* of 30 different serovars in Germany 2005–11. *J. Antimicrob. Chemother.* 68 1978–1981. 10.1093/jac/dkt163 23674765

[B24] El-Shatoury E. H. Azab El-Leithy M. Abou-Zeid M. A. El-Taweel G. E. El-Senousy W. M. (2015). “Antibiotic susceptibility of Shiga toxin producing *E. coli* O157:H7 isolated from different water sources,” in *Proceedings of the Biotechnology and Environmental Safety, at National Research Centre* Giza 10.2174/2210289201506010030

[B25] Enault F. Briet A. Bouteille L. Roux S. Sullivan M. B. Petit M.-A. (2017). Phages rarely encode antibiotic resistance genes: a cautionary tale for virome analyses. *ISME J.* 11 237–247. 10.1038/ismej.2016.90 27326545 PMC5315482

[B26] Eng S. K. Pusparajah P. Ab Mutalib N. S. Ser H. L. Chan K. G. Lee L. H. (2015). *Salmonella*: a review on pathogenesis, epidemiology and antibiotic resistance. *Front. Life Sci.* 8:284–293. 10.1080/21553769.2015.1051243

[B27] Feiner R. Argov T. Rabinovich L. Sigal N. Borovok I. Herskovits A. A. (2015). A new perspective on lysogeny: prophages as active regulatory switches of bacteria. *Nat. Rev. Microbiol.* 13 641–650. 10.1038/nrmicro3527 26373372

[B28] Ferreira M. R. Silva T. D. S. Stella A. E. Conceição F. R. Reis E. F. D. Moreira C. N. (2015). Detection of virulence factors and antimicrobial resistance patterns in Shiga toxin-producing *Escherichia coli* isolates from sheep. *Pesquisa Veterinária Brasileira* 35 775–780. 10.1007/s11250-012-0271-5

[B29] Food and Drug Administration [FDA] (2015). *Mecadox^®^ 10 Type A Medicated Article - Supplemental Approval (October 5 1998).* Available at: https://www.fda.gov/AnimalVeterinary/Products/ApprovedAnimalDrugProducts/FOIADrugSummaries/ucm064223.htm [Accessed May 9 2017].

[B30] Galland J. C. Hyatt D. R. Crupper S. S. Acheson D. W. (2001). Prevalence, antibiotic susceptibility, and diversity of *Escherichia coli* O157:H7 isolates from a longitudinal study of beef cattle feedlots. *Appl. Environ. Microbiol.* 67 1619–1627. 10.1128/AEM.67.4.1619-1627.2001 11282614 PMC92778

[B31] Graaf G. J. Jager L. P. Baars A. J. Spierenburg T. J. Vet Q. (1988). Some pharmacokinetic observations of carbadox medication in pigs. *Vet. Q.* 10 34–41. 10.1080/01652176.1988.9694143 3376409

[B32] Griffiths A. J. (2002). *Modern Genetic Analysis: Integrating Genes and Genomes.* New York, NY: Macmillan.

[B33] Guerra B. Soto S. Helmuth R. Mendoza M. C. (2002). Characterization of a self-transferable plasmid from *Salmonella enterica* serotype typhimurium clinical isolates carrying two integron-borne gene cassettes together with virulence and drug resistance genes. *Antimicrob. Agents Chemother.* 46 2977–2981. 10.1128/AAC.46.9.2977-2981.2002 12183256 PMC127424

[B34] Helms M. Ethelberg S. Mølbak K. Group D. T. S. (2005). International *Salmonella* Typhimurium DT104 infections, 1992-2001. *Emerg. Infect. Dis.* 11 859–867. 10.3201/eid1106.041017 15963280 PMC3367607

[B35] Herman K. M. Hall A. J. Gould L. H. (2015). Outbreaks attributed to fresh leafy vegetables, United States, 1973-2012. *Epidemiol. Infect.* 143 3011–3021. 10.1017/S0950268815000047 25697407 PMC4591532

[B36] Hunt J. M. (2010). Shiga toxin–producing *Escherichia coli* (STEC). *Clin. Lab. Med.* 30 21–45. 10.1016/j.cll.2009.11.001 20513540 PMC7126671

[B37] Imamovic L. Muniesa M. (2012). Characterizing RecA-independent induction of Shiga toxin2-encoding phages by EDTA treatment. *PLoS ONE* 7:e32393 10.1371/journal.pone.0032393 PMC3290563 22393404

[B38] Ishiguro N. Goto J. Sato G. (1980). Genetical relationship between R plasmids derived from *Salmonella* and *Escherichia coli* obtained from a pig farm, and its epidemiological significance. *J. Hyg.* 84 365–379. 10.1017/S0022172400026899 6762390 PMC2133900

[B39] Iweriebor B. C. Iwu C. J. Obi L. C. Nwodo U. U. Okoh A. I. (2015). Multiple antibiotic resistances among Shiga toxin producing *Escherichia coli* O157 in feces of dairy cattle farms in Eastern Cape of South Africa. *BMC Microbiol.* 15:213 10.1186/s12866-015-0553-y PMC4609099 26475706

[B40] Johnson K. E. Thorpe C. M. Sears C. L. (2006). The emerging clinical importance of non-O157 Shiga toxin-producing *Escherichia coli*. *Clin. Infect. Dis.* 43 1587–1595. 10.1086/509573 17109294

[B41] Jung Y. Jang H. Matthews K. R. (2014). Effect of the food production chain from farm practices to vegetable processing on outbreak incidence. *Microb. Biotechnol.* 7 517–527. 10.1111/1751-7915.12178 25251466 PMC4265071

[B42] Kapperud G. Gustavsen S. Hellesnes I. Hansen A. H. Lassen J. Hirn J. (1990). Outbreak of *Salmonella* typhimurium infection traced to contaminated chocolate and caused by a strain lacking the 60-megadalton virulence plasmid. *J. Clin. Microbiol.* 28 2597–2601. 2279988 10.1128/jcm.28.12.2597-2601.1990 PMC268240

[B43] Kenzaka T. Tani K. Sakotani A. Yamaguchi N. Nasu M. (2007). High-frequency phage-mediated gene transfer among *Escherichia coli* cells, determined at the single-cell level. *Appl. Environ. Microbiol.* 73 3291–3299. 10.1128/AEM.02890-06 17384307 PMC1907122

[B44] Kim H. H. Samadpour M. Grimm L. Clausen C. R. Besser T. E. Baylor M. (1994). Characteristics of antibiotic-resistant *Escherichia coli* O157:H7 in Washington State, 1984-1991. *J Infect Dis.* 170 1606–1609. 10.1093/infdis/170.6.1606 7996005

[B45] Köhler B. Karch H. Schmidt H. (2000). Antibacterials that are used as growth promoters in animal husbandry can affect the release of Shiga-toxin-2-converting bacteriophages and Shiga toxin 2 from *Escherichia coli* strains. *Microbiology* 146(Pt 5), 1085–1090. 10.1099/00221287-146-5-1085 10832635

[B46] Kropinski A. M. Sulakvelidze A. Konczy P. Poppe C. (2007). *Salmonella* phages and prophages—genomics and practical aspects. *Methods Mol. Biol.* 394 133–175. 10.1007/978-1-59745-512-1_9 18363236

[B47] Kurioka T. Yunou Y. Harada H. Kita E. (1999). Efficacy of antibiotic therapy for infection with Shiga-like toxin-producing *Escherichia coli* O157:H7 in mice with protein-calorie malnutrition. *Eur. J. Clin. Microbiol. Infect. Dis.* 18 561–571. 10.1007/s100960050348 10517193

[B48] Liakopoulos A. Geurts Y. Dierikx C. M. Brouwer M. S. Kant A. Wit B. (2016). Extended-spectrum cephalosporin-resistant *Salmonella enterica* serovar Heidelberg strains, the Netherlands. *Emerg. Infect. Dis.* 22 1257 10.3201/eid2207.151377 PMC4918182 27314180

[B49] Lin D. Chen K. Wai-Chi Chan E. Chen S. (2015). Increasing prevalence of ciprofloxacin-resistant food-borne *Salmonella* strains harboring multiple PMQR elements but not target gene mutations. *Sci. Rep.* 5:14754 10.1038/srep14754 PMC4648336 26435519

[B50] Majowicz S. E. Scallan E. Jones-Bitton A. Sargeant J. M. Stapleton J. Angulo F. J. (2014). Global incidence of human Shiga toxin-producing *Escherichia coli* infections and deaths: a systematic review and knowledge synthesis. *Foodborne Pathog. Dis.* 11 447–455. 10.1089/fpd.2013.1704 24750096 PMC4607253

[B51] Marinus M. G. Poteete A. R. (2014). High efficiency generalized transduction in *Escherichia coli* O157:H7. *F1000Res.* 2:7 10.12688/f1000research.2-7.v1 PMC3752737 24358856

[B52] Marti E. Variatza E. Balcázar J. L. (2014). Bacteriophages as a reservoir of extended-spectrum β-lactamase and fluoroquinolone resistance genes in the environment. *Clin. Microbiol. Infect.* 20 O456–O459. 10.1111/1469-0691.12446 24552593

[B53] Mclinden T. Sargeant J. M. Thomas M. K. Papadopoulos A. Fazil A. (2014). Component costs of foodborne illness: a scoping review. *BMC Public Health* 14:509 10.1186/1471-2458-14-509 PMC4041898 24885154

[B54] Meng J. Zhao S. Doyle M. P. Joseph S. W. (1998). Antibiotic resistance of *Escherichia coli* O157:H7 and O157:NM isolated from animals, food, and humans. *J. Food Prot.* 61 1511–1514. 10.4315/0362-028X-61.11.1511 9829195

[B55] Mitsuhashi S. (1993). Drug resistance in bacteria: history, genetics and biochemistry. *J. Int. Med. Res.* 21 1–14. 10.1177/030006059302100101 8319816

[B56] Mora A. Blanco J. E. Blanco M. Alonso M. P. Dhabi G. Echeita A. (2005). Antimicrobial resistance of Shiga toxin (verotoxin)-producing *Escherichia coli* O157:H7 and non-O157 strains isolated from humans, cattle, sheep and food in Spain. *Res. Microbiol.* 156 793–806. 10.1016/j.resmic.2005.03.006 15921895

[B57] Mueller-Doblies D. Speed K. Davies R. H. (2013). A retrospective analysis of *Salmonella* serovars isolated from pigs in Great Britain between 1994 and 2010. *Prev. Vet. Med.* 110 447–455. 10.1016/j.prevetmed.2013.02.023 23561958

[B58] Mulvey M. R. Boyd D. A. Olson A. B. Doublet B. Cloeckaert A. (2006). The genetics of *Salmonella* genomic island 1. *Microbes Infect.* 8 1915–1922. 10.1016/j.micinf.2005.12.028 16713724

[B59] Muniesa M. Colomer-Lluch M. Jofre J. (2013). Could bacteriophages transfer antibiotic resistance genes from environmental bacteria to human-body associated bacterial populations? *Mobile Genet. Elements* 3 739–751. 10.4161/mge.25847 PMC3812792 24195016

[B60] Nakaya R. Nakamura A. Murata Y. (1960). Resistance transfer agents in *Shigella*. *Biochem. Biophys. Res. Commun.* 3 654–659. 10.1016/0006-291X(60)90081-4 13727669

[B61] Noda T. Murakami K. Etoh Y. Okamoto F. Yatsuyanagi J. Sera N. (2015). Increase in resistance to extended-spectrum cephalosporins in *Salmonella* isolated from retail chicken products in Japan. *PLoS ONE* 10:e0116927 10.1371/journal.pone.0116927 PMC4314076 25642944

[B62] Ochiai K. Yamanaka T. Kimura K. Sawada O. (1959). Studies on inheritance of drug resistance between *Shigella* strains and *Escherichia coli* strains. *Nippon Iji Shimpo* 1861 34–46.

[B63] Rakhuba D. Kolomiets E. Dey E. S. Novik G. (2010). Bacteriophage receptors, mechanisms of phage adsorption and penetration into host cell. *Pol. J. Microbiol.* 59 145–155. 21033576

[B64] Ratnam S. March S. B. Ahmed R. Bezanson G. S. Kasatiya S. (1988). Characterization of *Escherichia coli* serotype O157:H7. *J. Clin. Microbiol.* 26 2006–2012. 3053758 10.1128/jcm.26.10.2006-2012.1988 PMC266806

[B65] Ridley A. Threlfall E. J. (1998). Molecular epidemiology of antibiotic resistance genes in multiresistant epidemic *Salmonella* typhimurium DT 104. *Microb. Drug Resist.* 4 113–118. 10.1089/mdr.1998.4.113 9650997

[B66] Riley L. W. Remis R. S. Helgerson S. D. Mcgee H. B. Wells J. G. Davis B. R. (1983). Hemorrhagic colitis associated with a rare *Escherichia coli* serotype. *N. Engl. J. Med.* 308 681–685. 10.1056/NEJM198303243081203 6338386

[B67] Ross J. Topp E. (2015). Abundance of antibiotic resistance genes in bacteriophage following soil fertilization with dairy manure or municipal biosolids, and evidence for potential transduction. *Appl. Environ. Microbiol.* 81 7905–7913. 10.1128/AEM.02363-15 26341211 PMC4616940

[B68] Sandvang D. Aarestrup F. M. Jensen L. B. (1998). Characterisation of integrons and antibiotic resistance genes in Danish multiresistant *Salmonella enterica* Typhimurium DT104. *FEMS Microbiol. Lett.* 160 37–41. 10.1111/j.1574-6968.1998.tb12887.x 9495010

[B69] Scallan E. Griffin P. M. Angulo F. J. Tauxe R. V. Hoekstra R. M. (2011). Foodborne illness acquired in the United States—unspecified agents. *Emerg. Infect. Dis.* 17:7–15. 10.3201/eid1701.091101p2 21192849 PMC3204615

[B70] Schmieger H. Schicklmaier P. (1999). Transduction of multiple drug resistance of *Salmonella enterica* serovar typhimurium DT104. *FEMS Microbiol. Lett.* 170 251–256. 10.1111/j.1574-6968.1999.tb13381.x 9919675

[B71] Schneider J. White P. Weiss J. Norton D. Lidgard J. Gould L. (2011). Multistate outbreak of multidrug-resistant *Salmonella* Newport infections associated with ground beef, October to December 2007. *J. Food Prot.* 74 1315–1319. 10.4315/0362-028X.JFP-11-046 21819658

[B72] Schroeder C. M. Zhao C. Debroy C. Torcolini J. Zhao S. White D. G. (2002). Antimicrobial resistance of *Escherichia coli* O157 isolated from humans, cattle, swine, and food. *Appl. Environ. Microbiol.* 68 576–581. 10.1128/AEM.68.2.576-581.2002 11823193 PMC126736

[B73] Seiffert S. N. Hilty M. Perreten V. Endimiani A. (2013). Extended-spectrum cephalosporin-resistant Gram-negative organisms in livestock: an emerging problem for human health? *Drug Resist. Updates* 16 22–45. 10.1016/j.drup.2012.12.001 23395305

[B74] Serra-Moreno R. Acosta S. Hernalsteens J. P. Jofre J. Muniesa M. (2006). Use of the lambda Red recombinase system to produce recombinant prophages carrying antibiotic resistance genes. *BMC Mol. Biol.* 7:31 10.1186/1471-2199-7-31 PMC1626079 16984631

[B75] Shousha A. Awaiwanont N. Sofka D. Smulders F. J. Paulsen P. Szostak M. P. (2015). Bacteriophages isolated from chicken meat and the horizontal transfer of antimicrobial resistance genes. *Appl. Environ. Microbiol.* 81 4600–4606. 10.1128/AEM.00872-15 25934615 PMC4551174

[B76] Smith H. O. Levine M. (1965). Gene order in prophage P22. *Virology* 27 229–231. 10.1016/0042-6822(65)90166-2 5840894

[B77] Smith S. M. Stocker B. A. (1966). Mapping of prophage P22 in *Salmonella* typhimurium. *Virology* 28 413–419. 10.1016/0042-6822(66)90053-5 18611473

[B78] Smith-Keary P. F. (1966). Restricted trandsuction by bacteriophage P22 in *Salmonella* typhimurium. *Genet. Res.* 8 73–82. 10.1017/S0016672300009927 5329975

[B79] Srinivasan V. Nguyen L. T. Headrick S. I. Murinda S. E. Oliver S. P. Larchmt N. (2007). Antimicrobial resistance patterns of Shiga toxin-producing *Escherichia coli* O157:H7 and O157:H7-from different origins. *Microb. Drug Resist.* 13 44–51. 10.1089/mdr.2006.9996 17536933

[B80] Suzuki D. T. Griffiths A. J. (2000). *An Introduction to Genetic Analysis*, 7th Edn. New York, NY: WH Freeman and Company.

[B81] Tanaka K. Nishimori K. Makino S.-I. Nishimori T. Kanno T. Ishihara R. (2004). Molecular characterization of a prophage of *Salmonella enterica* serotype Typhimurium DT104. *J. Clin. Microbiol.* 42 1807–1812. 10.1128/JCM.42.4.1807-1812.2004 15071057 PMC387611

[B82] Tanyashin V. I. Zimin A. A. Shliapnikov M. G. Boronin A. M. (2003). Transduction of plasmid antibiotic resistance determinants with pseudo-T-even bacteriophages. *Genetika* 39 914–926. 12942776

[B83] Thomas M. K. Murray R. Flockhart L. Pintar K. Fazil A. Nesbitt A. (2015). Estimates of foodborne illness-related hospitalizations and deaths in Canada for 30 specified pathogens and unspecified agents. *Foodborne Pathog. Dis.* 12 820–827. 10.1089/fpd.2015.1966 26259128 PMC4601674

[B84] Threlfall E. J. (2000). Epidemic *Salmonella* typhimurium DT 104–a truly international multiresistant clone. *J. Antimicrob. Chemother.* 46 7–10. 10.1093/jac/46.1.7 10882682

[B85] Lázaro N. S. Tibana A. Rodrigues D. P. Reis E. M. F. Quintaes B. R. Hofer E. (2004). Antimicrobial resistance and R-plasmid in *Salmonella* spp from swine and abattoir environments. *Pesqui Veterinria Bras.* 24 57–60. 10.1590/S0100-736X2004000200001

[B86] Vidovic S. Korber D. R. (2006). Prevalence of *Escherichia coli* O157 in Saskatchewan cattle: characterization of isolates by using random amplified polymorphic DNA PCR, antibiotic resistance profiles, and pathogenicity determinants. *Appl. Environ. Microbiol.* 72 4347–4355. 10.1128/AEM.02791-05 16751550 PMC1489585

[B87] Voetsch A. C. Gilder T. J. Angulo F. J. Farley M. M. Shallow S. Marcus R. (2004). FoodNet estimate of the burden of illness caused by nontyphoidal *Salmonella* infections in the United States. *Clin. Infect. Dis.* 38(Suppl. 3), S127–S134. 10.1086/381578 15095181

[B88] Volkova V. V. Lu Z. Besser T. Gröhn Y. T. (2014). Modeling the infection dynamics of bacteriophages in enteric *Escherichia coli*: estimating the contribution of transduction to antimicrobial gene spread. *Appl. Environ. Microbiol.* 80 4350–4362. 10.1128/AEM.00446-14 24814786 PMC4068684

[B89] von Wintersdorff C. J. Penders J. Van Niekerk J. M. Mills N. D. Majumder S. Van Alphen L. B. (2016). Dissemination of antimicrobial resistance in microbial ecosystems through horizontal gene transfer. *Front. Microbiol.* 7:173 10.3389/fmicb.2016.00173 PMC4759269 26925045

[B90] Walsh C. Duffy G. O’mahony R. Fanning S. Blair I. S. Mcdowell D. A. (2006). Antimicrobial resistance in Irish isolates of verocytotoxigenic *Escherichia coli* (*E. coli*)–VTEC. *Int. J. Food Microbiol.* 109 173–178. 10.1016/j.ijfoodmicro.2006.01.023 16626832

[B91] Watanabe T. Furuse C. Sakaizumi S. (1968). Transduction of various R factors by phage P1 in *Escherichia coli* and by phage P22 in *Salmonella* typhimurium. *J. Bacteriol.* 96 1791–1795. 4882025 10.1128/jb.96.5.1791-1795.1968 PMC315242

[B92] Weinbauer M. G. Rassoulzadegan F. (2004). Are viruses driving microbial diversification and diversity? *Environ. Microbiol.* 6 1–11. 14686936 10.1046/j.1462-2920.2003.00539.x

[B93] Werber D. Dreesman J. Feil F. Van Treeck U. Fell G. Ethelberg S. (2005). International outbreak of *Salmonella* oranienburg due to German chocolate. *BMC Infect. Dis.* 5:7 10.1186/1471-2334-5-7 PMC552305 15691371

[B94] Whichard J. M. Medalla F. Hoekstra R. M. Mcdermott P. F. Joyce K. Chiller T. (2010). Evaluation of antimicrobial resistance phenotypes for predicting multidrug-resistant *Salmonella* recovered from retail meats and humans in the United States. *J. Food Prot.* 73 445–451. 10.4315/0362-028X-73.3.445 20202328

[B95] Wing J. P. (1968). Transduction by phage P22 in a recombination-deficient mutant of *Salmonella* typhimurium. *Virology* 36 271–276. 10.1016/0042-6822(68)90144-X 4879190

[B96] Wong M. H. Chan E. W. Liu L. Z. Chen S. (2014). PMQR genes oqxAB and aac(6’)Ib-cr accelerate the development of fluoroquinolone resistance in *Salmonella* typhimurium. *Front. Microbiol.* 5:521 10.3389/fmicb.2014.00521 PMC4183184 25324840

[B97] World Health Organization [WHO] (2015). *WHO Estimates of the Global Burden of Foodborne Diseases.* Available at: http://apps.who.int/iris/bitstream/10665/199350/1/9789241565165_eng.pdf [Accessed May 9 2017].

[B98] Yang B. Qu D. Zhang X. Shen J. Cui S. Shi Y. (2010). Prevalence and characterization of *Salmonella* serovars in retail meats of marketplace in Shaanxi, China. *Int. J. Food Microbiol.* 141 63–72. 10.1016/j.ijfoodmicro.2010.04.015 20493570

[B99] Yosef I. Manor M. Kiro R. Qimron U. (2015). Temperate and lytic bacteriophages programmed to sensitize and kill antibiotic-resistant bacteria. *Proc. Natl. Acad. Sci. U.S.A.* 112 7267–7272. 10.1073/pnas.1500107112 26060300 PMC4466736

[B100] Zhang Y. Lejeune J. T. (2008). Transduction of *bla*_CMY -2_ *tet*(A), and *tet*(B) from *Salmonella enterica* subspecies *enterica* serovar Heidelberg to S. Typhimurium. *Vet. Microbiol.* 129 418–425. 10.1016/j.vetmic.2007.11.032 18187273

[B101] Ziebell K. Johnson R. P. Kropinski A. M. Reid-Smith R. Ahmed R. Gannon V. P. (2011). Gene cluster conferring streptomycin, sulfonamide, and tetracycline resistance in *Escherichia coli* O157:H7 phage types 23 45 and 67. *Appl. Environ. Microbiol.* 77 1900–1903. 10.1128/AEM.01934-10 21239555 PMC3067293

[B102] Ziebell K. Steele M. Zhang Y. Benson A. Taboada E. N. Laing C. (2008). Genotypic characterization and prevalence of virulence factors among Canadian *Escherichia coli* O157:H7 strains. *Appl. Environ. Microbiol.* 74 4314–4323. 10.1128/AEM.02821-07 18487402 PMC2493177

[B103] Zinder N. D. Lederberg J. (1952). Genetic exchange in *Salmonella*. *J. Bacteriol.* 64 679–699. 12999698 10.1128/jb.64.5.679-699.1952 PMC169409

